# Pathophysiology, Diagnostic Criteria, and Approaches to Type 2 Diabetes Remission

**DOI:** 10.7759/cureus.33908

**Published:** 2023-01-18

**Authors:** Abeer M Khamis

**Affiliations:** 1 Internal Medicine/Endocrinology, King Abdulaziz University, Jeddah, SAU

**Keywords:** the twin cycle, remission, diabetic ketoacidosis, t2dm, diabetes

## Abstract

Diabetes mellitus is a prevalent, life-threatening, and costly medical illness. Type 2 diabetes is defined by insulin resistance caused by persistent hyperglycemia, and it is frequently diagnosed by tests such as fasting blood glucose levels of more than 7.0 mmol/L or HbA1c values of more than 6.5%. Pathogenesis and development of type 2 diabetes mellitus are clearly varied, with genetic and environmental factors both leading to it. The attainment of glycated hemoglobin (HbA1c) levels below the diagnostic level and maintaining it for a minimum of six months without pharmacotherapy, is described as diabetes remission. Diagnosis is a two-part procedure. To begin, the diagnosis of diabetes must be confirmed, and then the type of diabetes must be determined. Even in patients who succeeded to maintain remission, follow-up with the physician and regular tests should be done to prevent any expected diabetes complications.

## Introduction and background

Diabetes mellitus is a prevalent, and costly medical illness. It affects one in every eleven persons globally and is associated with 11% of fatalities each year, spending USD 760 billion in direct costs alone. Diabetes is classified into type 1 diabetes, type 2 diabetes (T2D), gestational diabetes, and certain types of diabetes due to different causes.

T2D is considered the most common type of diabetes, causing about 90%-95% of all cases, and has been a fast-expanding global issue for decades. T2D is defined as insulin resistance caused by persistent hyperglycemia, and it is diagnosed based on plasma glucose tests as fasting blood glucose levels more than or equal to 7.0 mmol/L or HbA1c values more than or equal to 6.5% or two hours of plasma glucose during 75-gram oral glucose tolerance test equal or more than 11.1 mmol/L [1-3]. T2D mellitus (T2DM) affects more than 400 million people globally. Furthermore, Asia-Pacific is home to almost a third of these diabetic people. Because of the increased proportion of body fat and noticeable abdominal obesity, obese Asians have a greater chance of having T2DM than Caucasians having exactly the same BMI [4-6].

T2DM is categorized as a metabolic disease marked by two major physiological defects: resistance to insulin and beta cell malfunction, which do not arise simultaneously. Insulin resistance is described as a loss of sensitivity to insulin by target tissues, while beta cell dysfunction is described as inadequate insulin production by pancreatic beta cells to sustain normal glucose levels. Over time, the history of T2DM is marked by a steady degradation of beta cell activity [7,8].

Pathogenesis and development of T2DM are clearly varied, with genetic and environmental factors both leading to it. A genetic tendency to diabetes is usually present at birth; however, the hyperglycemia that identifies diabetes does not occur until adulthood, when it reaches the levels where it could be diagnosed. The availability of varied diets, the frequency of physical activity, the stress associated with family, employment, or other pressures, exposure to pollutants and poisons, and the availability of medical care are all factors that influence T2D expression. Pregnancy and glucocorticoid medication are two main cases that can cause hyperglycemia to appear earlier in vulnerable people.

As a result, patients may develop “gestational diabetes” or “steroid diabetes,” which are separate but similar disorders to ordinary T2D. Although glucose levels can recover to normal following pregnancy, there is still a higher risk of T2DM later [9-11].

Uncontrolled diabetes can cause complications such as myocardial ischemia, stroke, loss of vision, neuropathy, and kidney failure. The current goal of medical treatment is to reduce glucose levels, and manage of hypertension, dyslipidemia, and other cardiovascular risk factors in order to slow disease complications [12,13].

In the past few years, metabolic regulation therapies for T2D have vastly improved. When T2D is first diagnosed in adults, short-term pharmacological medication can often restore virtually standard glycemic control, allowing therapy to be stopped The best-studied reversal of “glucose toxicity” with the recovery of glycemic control is with early intense insulin therapy, but it can also happen with alternative interventions.GLP-1 receptor agonists and sodium-glucose co-transporter inhibitors, two new types of medicines, can achieve superior control of glucose levels with low likelihood to produce hypoglycemia [14-16]. In obese patients with T2D, bariatric surgery can enhance glycemic control and cardiovascular risk factors quickly [17,18]. Another scope of management is Weight loss for obese patients using different dietary approaches including therapeutic fasting which offers the ability to close the gap in diabetes management between medications and surgery by delivering similar calorie restriction and hormonal benefits to bariatric surgery without invasive surgery. Therapeutic fasting is described as a time of managed and voluntary withdrawal of all calorie-containing foods and beverages. Patients are advised to consume limitless amounts of very low-calorie fluids such as water, coffee, and bone broth during fasting times. To ensure appropriate micronutrients, a multivitamin supplement is recommended [19,20].

## Review

Diabetic remission

The attainment of glycated hemoglobin (HbA1c) levels underneath the diagnostic level and maintaining it for a minimum of six months without glucose-lowering medication, is described as diabetes remission. Remission is subsequently classified by Bus and coworkers into three main types; partial, complete, and prolonged where partial remission is characterized by HbA1c less than 6.5% and fasting glucose 5.6 to 6.9 mmol/L, which is maintained for at least one year in absence of medication. Complete remission is defined as a resumed normal glucose metabolism parameter with HbA1c less than 5.7% and fasting glucose less than 5.6 mmol/L without pharmacotherapy for at least one year. The term “prolonged remission” refers to remission that lasts longer than five years [21-23].

Another definition which focuses on achievement and maintenance of HbA1c less than 6.5% and fasting glucose less than 7 mmol on two occasions separated by at least six months after weight loss and discontinuation of all glucose-lowering medications was defined as remission by the Association of British Clinical Diabetologists (ABCD) and the Primary Care Diabetes Society (PCDS). This concept is practical, aligns with diabetes diagnosis criteria, and is simple to understand for both patients and physicians [24].

The discussion of the idea of T2DM remission, however, has recently become more pertinent, especially in light of two treatment breakthroughs: the effects of bariatric surgery and the outcomes of efficient weight reduction medications, as most recently demonstrated in the DiRECT experiment. The therapy typically results in remission in both situations. For example, in the DiRECT experiment, remission was reached in 85% of those patients with T2DM who obtained a weight loss of >15%. Similar outcomes are frequently observed following bariatric surgery, with weight loss reaching 40 kg in 1-2 years following surgery and a remission rate of 70%-80% [22].

There has not been much research done on how long-term remission affects late diabetic microvascular problems; however, there have been instances of regression following pancreas transplants and bariatric surgery. Following bariatric surgery, even pre-existing diabetic kidney disease has been found to improve. With long-term diabetic remission following bariatric surgery, the risk of macrovascular problems was decreased. However, during follow-up, a specific assessment of the development of late diabetes complications is required. Established issues also require ongoing evaluation [21,24].

The twin cycle hypothesis

The twin cycle concept suggests that excessive calorie consumption over time will transfer surplus energy storage in the form of triglycerides to the liver and other ectopic locations. Excess fat in the liver reduces the responsiveness of hepatocytes to insulin, resulting in hepatic insulin resistance and failure to shut off gluconeogenesis, resulting in high plasma glucose and insulin levels. In human and animal models, it has been shown that de-novo fatty acid synthesis contributes significantly to hepatic steatosis and that this is predominantly promoted by insulin [25,26].

A high baseline insulin level in T2DM will start a vicious cycle of hyperlipidemia and hyperglycemia. Beta-cells respond to hepatic insulin resistance in the early stages of T2DM development by boosting insulin secretion, elevating basal insulin levels, and reinforcing the liver cycle. Hepatic export of very low-density lipoprotein triglycerides (VLDL-TG) will rise under these conditions, raising circulating triglyceride levels [21]. Subcutaneous adipose tissue is a physiologically well-tolerated fat storage region; however, its storage capacity varies by individual. A personal fat threshold will be surpassed in the face of increased hepatic VLDL-TG export, resulting in ectopic fat deposition in the pancreas and other organs [27,28].

This will start the pancreatic cycle, in which toxic fat metabolites induce beta-cell malfunction in those who are vulnerable. Because of the compensating abilities of the beta-cell, this is tolerated in the early stages of disease progression. T2DM, on the other hand, emerges when beta-cells fail to adjust for increasing loss of functional mass.

Pathophysiology of T2D

According to current knowledge, the two key pathogenic causes that cause T2D, insulin resistance and beta cell dysfunction. Insulin resistance in skeletal muscles, liver, and pancreas may be caused by defects in glucose transporter and/or insulin signaling, while beta cell dysfunction is caused by oxidative stress, and high fatty acid content.

Insulin Resistance

Insulin resistance in the skeletal muscles is the first abnormality discovered in T2DM, and it is the primary cause of whole-body insulin resistance. Muscle insulin resistance's specific pathogenesis has yet to be discovered. Early improvements in fasting plasma glucose management, on the other hand, were shown to be primarily associated with improvements in liver insulin sensitivity. Despite having similar levels of muscular insulin resistance to those with T2D, many people are able to maintain normal blood glucose levels. Nonetheless, it has been established that long-term muscle insulin resistance induces elevated plasma insulin levels, which stimulate lipogenesis and accelerate fat formation in the liver. As a result, the pathophysiological significance of muscle insulin resistance plays out over a long period of time [29-31].

Hepatocytes are subjected to more metabolic stress when they eat a high-fat diet. Fatty acids can be produced by de novo lipogenesis within the hepatocyte. This fatty acid can be used to provide energy or mixed with glycerol to make triacylglycerols. Lipogenesis and synthesis of malonyl-CoA are inhibited in chronic hyper-insulinemia, preventing fatty acid transfer into mitochondria to be oxidized. As a result, newly produced triacylglycerol is guided toward accumulation or export, resulting in an increase in hepatic fat content. Furthermore, excess diacylglycerol disrupts the insulin receptor to insulin receptor substrate 1 (IRS-1) signaling pathway, resulting in insulin resistance. Excess fatty acids stimulate ceramide synthesis, which boosts gluconeogenic enzymes, resulting in more hepatic glucose generation. As a result, fasting plasma glucose levels rise [32-34].

Beta Cell Dysfunction

To maintain normoglycemia in the face of long-term insulin resistance, pancreatic beta cells increase the release of insulin from each cell. Diabetes develops over time as a result of insufficient compensation, such as insufficient insulin release from each cell or insufficient beta cell mass. Glucotoxicity and lipotoxicity are two acquired abnormalities thought to be linked to insulin secretion dysfunction. Chronic hyperglycemia uses up insulin secretory granules in beta cells, reducing the quantity of insulin available for secretion, resulting in glucotoxicity. Furthermore, it has been demonstrated that reducing glucose levels improves acute beta cell insulin response. Lipotoxicity is defined as a reduction in beta cell capability caused by long-term exposure to high levels of fatty acids [35,36].

Excess fatty acids restrict beta cell growth by inducing cell cycle inhibitors P16 and P18, which is exacerbated by high glucose levels, when compared to participants with normal glucose levels, T2DM patients have around half the amount of beta cells, indicating a reduced total potential insulin response. The loss of beta cells to apoptosis tends to increase with the duration of diabetes. Low-grade beta cell inflammation has been proposed as a contributing component in the development of T2DM [37,38].

Standard blood glucose test for diagnosing T2DM remission

As the key defining criterion, the group preferred HbA1c below the threshold typically used for first diagnosis of diabetes, 6.5% (48 mmol/mol), and maintaining at that level for at least three months without continuing the standard anti-hyperglycemic drugs. HbA1c measurement methods must be subjected to quality control, and assays must be standardized to standards matched with international reference values. A range of variables, such as variant hemoglobin, different rates of glycation, or abnormalities in erythrocyte survival that can occur in a variety of illness conditions, might impact HbA1c levels [39,40].

As a result, a normal HbA1c number may be present while glucose is really increased in some patients, or a high HbA1c value may be present when mean glucose is normal in others [41]. CGM can be used to analyze the association between mean glucose and HbA1c and discover trends beyond the usual range of normal in situations where HbA1c may be inaccurate or when the accuracy of HbA1c readings is unknown [42].

An FPG of less than 126 mg/dL (7.0 mmol/L) might be employed as an alternative criterion for remission. Testing two-hour plasma glucose after a 75-gram oral glucose challenge appears to be a less preferred option. Considering all options, the panel strongly recommended using HbA1c 6.5% (48 mmol/mol) as the most reliable, straightforward, and well-known defining criterion under normal conditions [43].

A diagnosis of remission can only be made when all glucose-lowering medicines have been discontinued for a period of time long enough to enable the drug's effects to fade and to examine the effect of the drug's absence on HbA1c readings. This standard would apply to all glucose-lowering medications, even those with other effects [42,43].

Strategies help to achieve T2DM remission

Pharmacological Interventions

Insulin: insulin treatment in DM appears to have a favorable effect by lowering insulin resistance, resulting in diabetes remission .patients where diet had no efficacy in lowering glucose level to the normal, insulin was given as a continuous S.C infusion in a physiological dose for two weeks to achieve normoglycemia, remission of diabetes was noted in nine out of 13 patients at six months In a sample of newly diagnosed diabetic patients in a Chinese population treated with continuous S.C insulin infusion for two weeks, Li et al. observed diabetes remission as well as improved beta cell activity [44].

Oral hypoglycemic agents: In recently diagnosed drug-naive diabetes patients with fasting blood sugar >200 mg, Gliclazide MR 60 mg was compared to 16 u of premixed insulin, combined with medical nutrition therapy. Despite the fact that blood glucose levels normalized in both groups in 2-6 weeks, drug-free remission with oral gliclazide was only 3% at six months, whereas it was 80% in the insulin-treated group (39). Oral hypoglycemic drugs (OHA) (glimepiride and/or metformin) vs. insulin glargine alone or in combination with the above OHAs were used to attain an FBS target of 6.1 mmol/L and a two-hour post meal glucose of 8 mmol/L, which was maintained for three months before therapy was halted. At 14%, the combination group (insulin + OHA) had a higher remission rate than the OHA group [45].

New drugs: Recent medications for diabetes feature a novel mechanism that results in significant weight loss. For instance, regardless of insulin secretion or insulin sensitivity, sodium-glucose cotransporter-2 (SGLT2) inhibitors lower blood sugar levels by inhibiting glucose resorption in the proximal convoluted tubule of the kidney. Because it is linked to urine glucose excretion, which results in caloric loss, this family of medications can reduce weight and visceral fat [46]. In a clinical trial that included SGLT2 inhibitors together with basal insulin and metformin as an intensive intervention, the group receiving SGLT2 inhibitors saw higher rates of remission than the traditional group (24.7% vs. 16.9%), and their risk of diabetes recurrence was cut by 43% [47].

The incretin hormone glucagon-like peptide-1 (GLP1) decreases the release of glucagon in the intestines; several GLP1 receptor agonists (GLP1-RAs) have been created and are now in use. When taken in early T2DM, liraglutide, a form of GLP1-RA, has been shown to help maintain pancreatic beta-cell activity. Tirozepatide, a medication that demonstrates dual activity in both GLP1 and glucose-dependent insulinotropic polypeptide (GIP), has recently been created and approved [48]. In a 52-week clinical study, the medication produced a remission rate ranging from 66% to 81%, depending on the drug dose. These medications have a significant weight reduction effect and a small risk of hypoglycemia episodes [49]. As a result, researchers might think about using these novel drugs instead of drastic measures with recognized health hazards like surgery, early intense insulin therapy, and VLCD. There is not enough concrete proof that these medications can cure diabetes, though, as they are relatively new treatments. More research is required to determine how new medications affect diabetes remission [50].

Metabolic Surgery

The effectiveness of bariatric surgery in achieving significant and long-term weight loss has been thoroughly shown in various trials; surgical methods produce much improved weight loss outcomes when compared to other medical treatments. Bariatric surgery results in significant improvements in obesity-related comorbidities, such as T2DM, in addition to long-term weight loss. Several research have focused on the metabolic effect of bariatric surgery since Dr. Pories presented metabolic surgery in 1995, presenting surgical treatments as a potential solution for diabetes [51]. Despite this, not all diabetic patients who have surgery obtain the desired result of diabetes remission. Nutritional concerns may arise as a result of surgery, necessitating long-term follow-up in patients. Candidates for metabolic surgery should be carefully chosen based on a thorough risk-benefit analysis performed prior to surgery [52].

Bariatric surgery, when used particularly to treat diabetes, considerably improves glycemic control and lowers cardiovascular risk factors, according to randomized, controlled trials, which have largely been short-term studies. it was reported in the Surgical Treatment and Medications Potentially Eradicate Diabetes Efficiently (STAMPEDE) trial that, at one year and three years after randomization, both gastric bypass and sleeve gastrectomy were better compared to medications alone in terms of achieving excellent glycemic control (i.e., glycated hemoglobin 6.0%), lowering cardiovascular risk, better quality of life and lowering drug therapy use [53-58].

T2D remission rates of 55% to 95% have been reported in observational studies of bariatric surgeries. A nonrandomized, prospective trial comparing bariatric surgery to traditional obesity treatment found that surgery had greater diabetes remission rates after two and ten years, but that recurrence was gradual. A single previous randomized, controlled trial compared medical therapy to gastric banding in patients with moderate-to-severe obesity (BMI 30 to 40), however it only included patients with early diabetes (less than two years). Gastric banding outperformed medical therapy in terms of glycemic control and weight loss in that trial. Patients in the STAMPEDE trial, on the other hand, had more advanced T2D, with an average disease duration of more than eight years and a mean baseline glycated hemoglobin level of 8.9% to 9.5%, while being treated with about three diabetes medications, including insulin. The STAMPEDE trial's inclusion of patients with more advanced T2D likely explains the lower percentage of diabetes remission seen; other variations from earlier trials included less severe obesity [59,60].

Predictor models of DM remission after bariatric surgery; Lee et al. proposed the ABCD diabetic surgery score in a publication published in 2012, which is made up of four independent predictors of T2DM remission: patient age, BMI, C-peptide level, and diabetes duration. T2D remission rates are projected to be greater in individuals with higher scores. Another scoring system is individualized metabolic surgery (IMS) score, which divides patients into three stages of diabetes severity. It includes four risk factors: pre-op number of diabetes medications, pre-op insulin use, pre-op duration of diabetes (years), pre-op glycemic control (<7%) and T2DM remission rate is expected to be higher after metabolic surgery in patient with mild severity [61,62].

Dietary Intervention

According to the DiRECT trial, there were many measures that were predictors of remission, some of which were not clarified by variations in weight loss; however, remission can be achieved efficiently and properly using weight management, and most people will achieve remission with a 10 kg/percent weight loss, though a 15 kg/percent weight loss offers better confirmation [63,64]. Remission persistence is linked to weight loss maintenance, with relapsers gaining more weight between 12 and 24 months than those who remain in remission. Weight regains was highly linked to re-accumulation of ectopic fat inside the liver and pancreas in DiRECT patients who had achieved remission but then relapsed. These findings support the theory that T2D onset and remission are governed by surpassing or falling below a “personal fat threshold” within the liver and pancreas [65,66].

Low-fat vs. low-carbohydrate diets have been a point of contention for a long time, when low-fat diets were the norm. This was due to the fact that fat has a larger calorie density than carbohydrate, as well as worries about the cardiovascular dangers of high-fat diets. Randomized trials in general populations have shown that both low calorie and low carbohydrate diets can help people lose weight if they stick to them [67-69].

Low carbohydrate diets show somewhat greater weight loss up to one year than low fat diets, with a tiny difference of roughly 1 kg body weight, but there is no difference after two years, according to the limited randomized trial evidence. Another study with long-term follow-up found that a very low carbohydrate diet can help persons with T2D lose 12 kg and keep it off for two years. Furthermore, a two-year follow-up of a cohort in a single British general practice found that after two years on a low-carbohydrate diet, the median weight loss was 8.3 kg [70].

It is almost unheard of to adopt a therapeutic fasting diet for T2D treatment. A case study found that fasting for 24 hours can reduce or even eliminate the need for diabetes medication. As recently revealed in an open-label Diabetes Remission Clinical Trial, calorie restriction and weight loss are crucial determinants in T2D remission (DiRECT). However, very few studies or examples of therapeutic fasting as a cure for T2D have been documented or published so far [71].

A study found that starting a therapeutic fasting program removed the requirement for insulin in all three individuals. Within a month, all three patients were successful, one in as little as five days. In addition, all patients saw improvements in a variety of clinically important health outcomes, including HbA1C, BMI, and waist circumference [71]. This decrease in risk factors will almost certainly lower the likelihood of subsequent difficulties. Wing et al. discovered that modest weight loss of 5%-10% was linked to significant changes in cardiovascular disease risk variables in T2D patients [72,73].

Monitor and Follow-up

A remission is a situation in which diabetes is not present, but it still needs to be monitored because hyperglycemia can resurface. Recurrence of T2D can be caused by weight increase, stress, or a continued deterioration in ß-cell function. HbA1c testing or another diagnostic tests should be done at least once a year. Pharmacotherapy for other illnesses that contain medications that induce hyperglycemia, such as glucocorticoids and certain antipsychotics, should be avoided. The basic consequences of diabetes, such as retinopathy, nephropathy, neuropathy, and an increased risk of cardiovascular disease, might still develop even after a remission. People in remission should be encouraged to have regular retinal screening, renal function testing, foot evaluations, and blood pressure and weight measurements, in addition to continued HbA1c monitoring [74,75].

Individuals in remission should be recommended to continue to be monitored by their doctors, with regular checks. Following a quick fall in glucose levels after a lengthy period of hyperglycemia, there is a risk of developing microvascular disease. When glycemic control is inadequate and retinopathy is present, rapid glucose reductions should be prevented, and retinal screening should be repeated [76].

Duration of T2DM Remission

The direct effects of pharmacotherapy do not remain when a remission is documented following transient use of glucose-lowering medications. Reversal of the negative effects of poor metabolic control on insulin secretion and action may result in a remission, but other underlying abnormalities remain, and the length of the remission varies. When a consistent adjustment in lifestyle leads to remission, however, changes in food consumption, physical exercise, and stress and environmental factor management can favorably modify insulin secretion and action over time. Long-term remissions are conceivable, but not guaranteed, in this scenario. The consequences of metabolic surgery are more severe and, in general, last longer [77,78].

There is no information on the long-term impact of remission on mortality, cardiovascular events, functional ability, or quality of life. The metabolic and clinical mechanisms that influence these outcomes during remission are largely unknown. The length of a remission generated by various therapies is yet unknown, and variables linked to recurrence from remission should be investigated further.

## Conclusions

T2DM is a chronic metabolic disorder characterized by high glucose levels either due to loss of beta cell function that secretes insulin or resistance to the produced insulin. Good management of this condition is necessary as it can lead to other complications such as retinopathy, neuropathy, and cardiovascular disorders. Keeping the blood glucose level below the diagnostic level for at least three months without medications is described as diabetic remission, this could be achieved by hypoglycemic medications, dietary intervention, and metabolic surgery. The last two interventions are used mainly in overweight or obese diabetic patients, where changing lifestyle can eliminate the need for medications through significant weight loss, even in patients who succeeded to maintain remission, follow-up with the physician and regular tests should be done to prevent any expected diabetes complications.
